# All the workplace is a stage: extending the double empathy problem through performative relationality

**DOI:** 10.3389/fpsyg.2026.1893333

**Published:** 2026-07-14

**Authors:** Nicole M. Reed, Timothy J. Vogus

**Affiliations:** 1 Scheller College of Business, Georgia Institute of Technology, Atlanta, GA, United States; 2 Owen Graduate School of Management, Vanderbilt University, Nashville, TN, United States

**Keywords:** double empathy problem, emotion regulation, emotional labor, expectancy violations, neurodiversity at work, neuroinclusive workplaces, performative relationality, workplace masking

## Abstract

Neurodivergent adults frequently experience difficulties in obtaining and sustaining employment due to challenges building and maintaining workplace relationships. Milton’s double empathy problem holds that relational breakdowns in mixed-neurotype interactions occur not because of a social deficit within the neurodivergent interactional partner, but rather because of a mutual misalignment in social expectations and behaviors. A core implication of the double empathy problem is that shared-neurotype dyads experience smoother communication and shared understanding than mixed-neurotype dyads. However, recent work has questioned this claim and the double empathy problem, generally. We address this critique by arguing that it reveals the power of neurotypical expectations and that even within-neurotype interactions often adhere to these expectations to avoid the emotional and social costs of violating them. We draw upon expectancy violations theory as well as emotional regulation and labor theories to develop the concept of *performative relationality*—a chronic, risk-responsive form of social performance in response to social and evaluative threats through which neurodivergent individuals regulate emotion and expression to reduce anxiety and adhere to neurotypical norms. Using a vignette from Nicole’s lived work experience, we illustrate how performative relationality can lead to mutual misunderstanding in same neurotype dyads by making negative expectancy violations feel risky. We further detail how expectancy violations and performative relationality necessitate interventions to (re)shape norms and expectations to reduce pressure to engage in performative relationality due to fear of social and performance sanctions. We close by discussing future research to examine these interventions and their effects.

## 1 Introduction

Neurodivergent individuals face a disproportionate number of employment barriers rooted in relational expectations rather than task competence (LeFevre-Levy et al., 2023; Hayward et al., 2020). These barriers center on neurotypical norms, or socially constructed interactional standards, rooted in non-neurodivergent processing styles (Doyle, 2020), which employees must continuously mimic to be perceived as a “good fit” (Hayward et al., 2020). Pressuring neurodivergent employees to adapt to neurotypical norms inherently reinforces stigmatizing assumptions that neurodivergence is a deficit (LeFevre-Levy et al., 2023).

In response to the persistence of such stigma, the double empathy problem (Milton, 2012) reconceptualizes social breakdowns between neurodivergent and neurotypical people as mutual misunderstanding that results from a breakdown *between* both parties rather than one-sided social deficits. More specifically, the double empathy problem posits that cross-neurotype breakdowns in understanding result from individual differences in attention, communication, interpretation, and sensory experience associated with neurodivergence and neurotypicality. In parallel, the double empathy problem responds to assertions of autistic (and more broadly neurodivergent) deficits of empathy by asserting that there is a greater likelihood of empathic understanding in within-neurotype interactions. This claim has been subject to recent criticism, namely that in prior experimental studies shared-neurotype dyads do not reliably show smoother communication and greater mutual understanding (Livingston et al., 2024). However, Kilgallon et al. (2026) assert that Livingston et al. (2024) have mischaracterized the double empathy problem by ignoring that “this is not to say that autistic people will automatically be able to connect and feel empathy with other autistic people they meet any more than two random non-autistic people would” (Milton et al., 2022, p. 1901). We argue that disconnects within autistic dyads reveal the power of neurotypical expectations and illustrate how even within-neurotype interactions often adhere to these expectations to avoid the emotional and social costs of violating them (Burgoon and Le Poire, 1993; Burgoon, 2015). We refer to this process as *performative relationality*—a chronic, risk-responsive form of social performance through which neurodivergent individuals regulate emotion and expression to adhere to neurotypical norms. That is, performative relationality involves not merely “fitting in,” but also engaging in emotional regulation and labor under evaluative threat as a survival mechanism (Kidwell et al., 2023; Côté, 2005).

Drawing upon expectancy violations theory, we identify chronic and pervasive relational barriers in neuronormative workplaces that induce performative relationality that focuses on minimizing evaluative and interpersonal risk (i.e., avoiding negative expectancy violations) even when interacting with other neurodivergent colleagues. In doing so, we detail the conditions under which within-neurotype interactions may follow similar patterns to cross-neurotype interactions. Our focus on the emotional underpinnings of performative relationality spotlights the importance of the emotional dimension of the double empathy problem and its consequences. To illuminate these conceptual ideas in practice, we provide a vignette from Nicole’s lived experience at work. We close with practices that address the underlying neurotypical norms and foster less performative, higher quality workplace relationships both within and across neurotypes.

## 2 Expectancy violations theory and the double empathy problem

Expectancy violation theory focuses on enduring cognitions regarding the anticipated behavior of others (i.e., expectations) that are shaped by personal, relational (e.g., trust), and contextual (e.g., social norms) factors (Burgoon and Le Poire, 1993). Violations occur when people behave or communicate in ways inconsistent with others’ expectations and are experienced as psychologically arousing and subsequently activate a cognitive-evaluative process that results in valencing the violation as positive or negative (Burgoon, 2015). Generally, behaviors signaling decreased involvement (avoiding eye contact, leaning away, and adopting an indirect body orientation), which are common neurodivergent behaviors, are interpreted as negative expectancy violations that signify disinterest or disrespect (Burgoon and Le Poire, 1993). In turn, they are associated with reduced attraction, credibility, and perceptions of how rewarding the interaction is. In addition to nonverbal factors, *how* information is communicated can also result in negative expectancy violations as when a neurodivergent coworker’s succinct response is perceived as “too blunt” instead of the more neutral “direct” or a positive “clear and efficient.” These interpretations of proxemics (i.e., social norms regarding space and distance), other forms of nonverbal communication, and interaction style and content are shaped by workplace (neurotypical) norms. As we describe next, the double empathy problem reflects negative expectancy violations from the neurotypical baseline in interpersonal interactions.

The double empathy problem conceptualizes cross-neurotype breakdowns in understanding as mutual and grounded in differences in attention, communication, interpretation, and sensory experiences (Williams et al., 2021). But the double empathy problem is also socially constructed in that there is assumed to be a shared baseline for how to interact and that deviations that result in social misunderstanding from the shared baseline (i.e., expectancy violations) are the fault of the neurodivergent person (Vogus, 2025). What is less directly stated in the conceptualization of the double empathy problem is the emotional burden created when neurodivergent employees anticipate that their behaviors will be measured and judged against these expectations. Specifically, there is a persistent fear of violating social norms and expectations. That leads to actions, like masking (Pearson and Rose, 2023), that may ostensibly bridge the double empathy problem as a neurodivergent person tries to enact neurotypical social norms, but at great cost to the neurodivergent person (Raymaker et al., 2020). Masking in cross-neurotype interactions leads to lower quality interactions and relationships because both parties are not seen as they want to be seen and the relationship does not benefit from the unique experience and expertise of each person as (at least) one party is self-censoring. By “self-censoring,” we refer not only to suppressing natural behavior, but also actively performing behaviors or interactional styles that one would not otherwise enact. As a result, the interaction lacks the vitality and mutual responsiveness characteristic of high-quality connections (Dutton and Heaphy, 2003). We argue that the same dynamics are likely to unfold in within-neurotype (including neurodivergent-neurodivergent) interactions in social contexts where neurotypical interaction styles are expected. As a result, employees must continually manage how their intentions, emotions, and social behavior will be read by their coworkers. We refer to the emotional labor and regulation people engage in to avoid violating expectations as *performative relationality*. Although appearing to reduce social misalignment, performative relationality amplifies the double empathy problem. It does so by creating greater social and empathetic distance even when both interaction partners are neurodivergent.

When applied specifically to autism, heterogeneity in presentation can push people to engage in performative relationality because while there is assumed similarity within-neurotype in terms of attention, communication, and sensory experience, there is still significant variation (Bury et al., 2019). As a result, it creates uncertainty and anxiety about the interaction, especially in a setting where neurotypical norms of communication and expression are likely to be pervasive (e.g., the workplace).

Heterogeneity across forms of neurodivergence (e.g., autism, ADHD, dyslexia) should be theorized in a manner consistent with Milton’s (2012) initial formulation of differently disposed social actors with different experiences. That is, assuming that each party to the interaction acts with corresponding differences in attention, communication, and sensory experiences. As such, cross-neurotype interactions among neurodivergent people are likely to resemble the canonical autistic-non-autistic interaction underlying the double empathy problem. Therefore, we would expect something closer to masking whereby one does not reveal their neurodivergence and actively tries to hide or suppress it. We argue this is especially likely when neurotypical norms are especially strong and absent specific interventions to make disclosure easier. However, this merits further empirical exploration.

## 3 Performative relationality as a mechanism of mutual misalignment

We suggest that fear of negative expectancy violations and the masking that follows may explain why prior tests of the double empathy problem have failed to find that within-neurotype interactions yield higher levels of mutual understanding. In other words, the fear of violating expectations extends across interactions, especially in the workplace. We argue that this occurs for two reasons. First, non-disclosure, meaning one does not know with certainty who is neurodivergent (or not). Second, even when the other party is also known to be neurodivergent, embracing neurodivergent approaches to sociability (Rosqvist, 2019) may be seen as risky and counternormative (e.g., violates norms regarding proxemics, non-verbal, and verbal communication, Burgoon and Le Poire, 1993). This is especially likely in contexts with strong neurotypical cultures like business schools and workplaces (Spoor et al., 2026).

Neurodivergent people internalize these norms and expectations regarding how individuals express emotions, interpret social signals, and develop strategies to regulate relational risks and reduce anxiety. Specifically, sustained performative relationality entails engaging in the proxemics, nonverbal, and verbal practices of the neuronormative expectations. Importantly, because performative relationality is a chronic adaptive strategy acquired through repeated exposure to social threat, it may become a habitual, safety-oriented response rather than a purely context-sensitive relational choice. Consequently, it does not necessarily “turn off” when both interaction partners are neurodivergent: even in neurodivergent-neurodivergent interactions, individuals may continue to default to performative relationality because they remain wary of evaluation and prioritize risk management. As such, the double empathy problem can persist even in within-neurotype interactions.

Although performative relationality draws upon masking and camouflaging, surface acting, and impression management, it remains distinct from its neighboring concepts. Masking and camouflaging describe how people alter or suppress their neurodivergent responses and behaviors to navigate social situations and conform to neurotypical expectations and to even “pass” as neurotypical (Hull et al., 2017; Pearson and Rose, 2023). In other words, it is focused on personal identity protection. Similarly, surface acting refers to how individuals alter their outward emotional expressions to comply with organizational social norms across contexts (Grandey, 2000), while impression management broadly describes efforts to protect how one is perceived by others (Bolino et al., 2008). Performative relationality instead captures how a chronically embedded protective orientation is enacted and interpreted within the particulars of a specific interaction, with each interaction partner serving as both audience and actor on the social stage. Each person enters the interactional stage with expectations about appropriate behavior that are shaped by personal experience, the relationship, and the broader neuronormative context. Their self-presentational strategies are then observed and evaluated by the other person. When both partners display different relational performances and evaluate the other’s behavior against incompatible expectations, each may perceive the other’s actions as a negative expectancy violation. Performative relationality therefore explains how individual protective efforts to avoid or mitigate judgment can become reciprocal expectancy violations that produce mutual misunderstanding and relational strain. As such, performative relationality operates more as sensegiving (Gioia and Chittipeddi, 1991)—presenting information in a manner that it is understood and avoids social sanctions. But performative relationality is fundamentally relational. As in the vignette that follows, when interaction partners display different performances or ostensibly interpret one another’s behaviors through conflicting relational expectations, these performances may become two-sided expectancy violations that produce misunderstanding and relational strain.

Performative relationality is grounded directly in Goffman’s dramaturgical framework in three primary ways. First, Goffman (1959) views social interaction as a type of performance, whereby individuals manage their “front-stage” behavior to externalize a desired identity and maintain a shared social understanding of the situation. In parallel, foundational work on performativity (e.g., Derrida, 1979; Butler, 1993) focuses on how identities are mutually constituted through discursive acts. This performance parallels the behaviors that neurodivergent employees engage in at the workplace when they regulate emotional expression, body language, and interaction style to align with neuronormative workplace standards and enact an identity as a member of the organization. Second, Goffman (1959) identified a salient distinction between what people intend to express and what they unintentionally convey. In the context of the workplace, a behavior meant to signal professionalism or warmth might instead be interpreted by the other interaction partner as coldness or disrespect. Third, performative relationality applies the same logic of Goffman’s (1963) work on stigma. Neurodivergent employees, who are individuals with potentially stigmatized identities, anticipate judgment and alter their behaviors to mitigate risk and penalty in the workplace. Our framework of performative relationality underscores that neuronormative pressures contribute to the recurring chronic and safety-oriented nature of these social performances within interactions.

## 4 Workplace vignette: neurodivergent-neurodivergent dyadic interaction

The following vignette illustrates how performative relationality in a within-neurotype dyad can generate relational misalignment. The vignette is based upon Nicole’s experience of an actual workplace interaction. Descriptions of her own intentions and interpretations are first-person accounts, whereas explanations of her coworker’s motives represent theoretical interpretations of his observable behavior rather than verified claims about his internal experience.

Nicole, an autistic professional working on an analytics team, meets with another autistic coworker to figure out why data on their shared dashboard is inconsistent with company performance results as they are getting ready for their organization’s most important annual meeting with executives. Based on previous work experience, Nicole has learned that meetings tend to go more smoothly when she leads with interpersonal warmth and amicability. Before discussing the technical issues at hand, Nicole attempts to soften her tone, make some light personal conversation, and create a friendly social environment. However, Nicole’s colleague is currently experiencing significant performance pressure and therefore has chosen to manage his job-related risks differently. In order to prevent speaking incorrectly, generating confusion, or delaying production due to something he says, he responds to small talk with short, one-word answers, immediately addresses the issue, and remains focused solely on the task.

As the interaction unfolds, Nicole interprets her coworker’s lack of small talk, minimal responses, and limited follow-up engagement with the task as signals of dismissiveness, interpersonal bias, and lack of care about the work. Conversely, her coworker may interpret Nicole’s efforts to sustain an amicable tone, ask follow-up questions, and revisit points of clarification as inefficient and accusatory. The exchange therefore becomes increasingly strained, and both leave with the impression that the other has acted disrespectfully and inappropriately to one another.

While both Nicole’s and her coworker’s self-presentation strategies are attempts to manage relational risks in the workplace, each is grounded in different interpretations of the contextual, relational, and personal expectations and produces differing baselines for discerning a negative expectancy violation (Burgoon, 2015). These two manifestations of performative relationality are employed to protect against adverse social and workplace ramifications in organizations with prevailing neurotypical expectations (Pryke-Hobbes et al., 2023; Hull et al., 2017).

This vignette further illuminates both the way in which the double empathy problem can manifest and escalate in within-neurotype interactions as well as the multiple ways in which performative relationality is enacted. Specifically, both individuals engage in performative relationality but employ different strategies due to different assumptions about what is needed for the situation. These strategies include compensatory interaction scripting, communication shaping, emotional regulation, suppression of sensory or behavioral expressions, and disclosure management (Jones and King, 2014; Côté, 2005; Grandey, 2000). Performing in accordance with predominant organizational norms, individuals must continually regulate emotional expression by suppressing some responses, amplifying others, and monitoring how those displays will be received by interaction partners. Therefore, performative relationality is the behavioral manifestation of ongoing regulatory effort: a form of emotional labor and emotion regulation through which employees manage feeling, expression, and interpersonal risk in real time (Côté, 2005; Grandey, 2000).

## 5 Discussion

The double empathy problem offers a powerful lens for understanding neurodivergent experiences in neuronormative contexts. However, prior empirical work has been criticized for failing to support fundamental tenets of the double empathy problem, namely, that within-neurotype interactions are smoother and generate greater understanding between interaction partners. We integrated expectancy violations theory and developed the concept of performative relationality to understand why mutual misunderstanding may persist even in within-neurotype interactions. Specifically, the expectations emanating from powerful neurotypical social norms and the social and emotional pressure that accompany them offer a more comprehensive account of neurodivergent-neurodivergent communication breakdowns. Expectancy violations theory illustrates how neurotypical norms become the evaluative baseline against which interaction quality is measured, while performative relationality explains how neurodivergent individuals engage in emotional regulation and labor to not negatively violate that baseline. Together, expectancy violations and performative relationality point to the necessity of intervening at the level of norms and expectations to reduce pressure to engage in performative relationality due to fear of social and performance sanctions. We explore these implications next.

### 5.1 Practical implications

Because performative relationality is a function of neuronormative expectations, efforts to revise those expectations may be helpful in managing the double empathy problem. For example, Longmire et al. (2025) point to normalizing variance in patterns of interrelating to broaden the range of behaviors deemed acceptable and lessen the risks and social costs of negative expectancy violations. Similarly, viewing differences in interpersonal approach as value-added contributions may also lessen pressures for performative relationality (Ezerins et al., 2023). At the dyadic level, tools such as “user manuals” or co-created relational agreements can enable interaction partners to share their personal preferences and establish interpersonal expectations without requiring disclosure. These practices could also lessen the perceived need for performative relationality and enhance mutual understanding.

### 5.2 Future research and boundary conditions

While our framework draws on research across neurodivergent populations, much of the empirical base for masking comes from autism research. The vignette, though grounded in lived experience, is illustrative and does not constitute empirical validation. As noted above, future experimental research that manipulates expectations in the form of interpersonal and/or organizational norms to test whether more (neuro)inclusive approaches would yield more cross- and within-neurotype mutual understanding. In addition, performative relationality may be perceived as less necessary if there is a greater degree of psychological safety between those interacting (e.g., prior positive relational history and trust) or if third parties create less threatening conditions (e.g., job coaches, leaders, human resource professionals). All these merit further conceptual and empirical exploration. To further unpack another dynamic in Nicole’s vignette, future work could also explore the intersection of gender and neurotypical expectations (see also Ezerins et al., 2026) in shaping how individuals engage in performative relationality.

More generally, although we argue that performative relationality can explain within-neurotype breakdowns in understanding, it is worth noting that our arguments need to be subjected to empirical testing while accounting for alternate explanations. Beyond the specific uncertainty and anxiety regarding a workplace interaction, differences in generalized levels of anxiety could also explain breakdowns and mutual misunderstanding in within-neurotype interactions. Similarly, differences in co-occurring conditions or sensory needs could also explain within-neurotype breakdowns (Masi et al., 2017). Empirical explorations of performative relationality should account for these alternate explanations. Moreover, when neuronormative workplace pressures are lessened, and within-neurotype breakdowns are observed, performative relationality should not be treated as the primary explanation for why it occurs. Lastly, careful task design and methodological rigor are essential for testing the double empathy problem and its implications (Crompton et al., 2025).

### 5.3 A neuroinclusive future

Advancing neuroinclusivity in the workplace requires more than a company’s blanket invitation for employees to “be themselves.” The double empathy problem should be understood not as purely cognitive, but as a relationally embedded and emotional response to neuronormative pressures. Specifically, pressures to avoid negative expectancy violations regarding the individual, the relationship, and the context. Therefore, we propose intervening to broaden relational expectations to help reduce the likelihood and threat of violating them and rigorously exploring these ideas empirically. Ultimately, a neuroinclusive future requires workplaces that no longer privilege neurotypicality as the standard for acceptable behavior but instead broaden the workplace stage so that diverse forms of interaction can truly foster mutual understanding.
